# Novel *IRF6* mutations in families with Van Der Woude syndrome and popliteal pterygium syndrome from sub-Saharan Africa

**DOI:** 10.1002/mgg3.66

**Published:** 2014-01-27

**Authors:** Azeez Butali, Peter A Mossey, Wasiu L Adeyemo, Mekonen A Eshete, LauRen A Gaines, Dee Even, Ramat O Braimah, Babatunde S Aregbesola, Jennifer V Rigdon, Christian I Emeka, Olutayo James, Mobolanle O Ogunlewe, Akinola L Ladeinde, Fikre Abate, Taye Hailu, Ibrahim Mohammed, Paul E Gravem, Milliard Deribew, Mulualem Gesses, Adebowale A Adeyemo, Jeffrey C Murray

**Affiliations:** 1 Department of Oral Pathology, Radiology and Medicine, College of Dentistry, University of IowaIowa City, Iowa; 2 Department of Orthodontics, University of DundeeDundee, Scotland; 3 College of Medicine, University of LagosLagos, Nigeria; 4 Addis Ababa UniversityAddis Ababa, Ethiopia; 5 Obafemi Awolowo UniversityIle-Ife, Nigeria; 6 Center for Research on Genomics and Global Health, National Human Genome Research Institute, National Institutes of HealthBethesda, Maryland

**Keywords:** *IRF6*, popliteal pterygium syndrome, sub-Saharan Africa, Van der Woude syndrome

## Abstract

Orofacial clefts (OFC) are complex genetic traits that are often classified as syndromic or nonsyndromic clefts. Currently, there are over 500 types of syndromic clefts in the Online Mendelian Inheritance in Man (OMIM) database, of which Van der Woude syndrome (VWS) is one of the most common (accounting for 2% of all OFC). Popliteal pterygium syndrome (PPS) is considered to be a more severe form of VWS. Mutations in the *IRF6* gene have been reported worldwide to cause VWS and PPS. Here, we report studies of families with VWS and PPS in sub-Saharan Africa. We screened the DNA of eight families with VWS and one family with PPS from Nigeria and Ethiopia by Sanger sequencing of the most commonly affected exons in *IRF6* (exons 3, 4, 7, and 9). For the VWS families, we found a novel nonsense variant in exon 4 (p.Lys66X), a novel splice-site variant in exon 4 (p.Pro126Pro), a novel missense variant in exon 4 (p.Phe230Leu), a previously reported splice-site variant in exon 7 that changes the acceptor splice site, and a known missense variant in exon 7 (p.Leu251Pro). A previously known missense variant was found in exon 4 (p.Arg84His) in the PPS family. All the mutations segregate in the families. Our data confirm the presence of *IRF6*-related VWS and PPS in sub-Saharan Africa and highlights the importance of screening for novel mutations in known genes when studying diverse global populations. This is important for counseling and prenatal diagnosis for high-risk families.

## Introduction

Orofacial clefts (OFC) are complex genetic traits that are often classified as syndromic or nonsyndromic clefts. To date, over 500 syndromic clefts have been identified and included in the Online Mendelian Inheritance in Man (OMIM) database (http://www.omim.org), and for many of these the underlying genetic factors have been identified. Van der Woude syndrome (VWS) (OMIM:119300) is an autosomal dominant disorder affecting 1/40,000 people worldwide (Lam et al. 2010) and also one of the most common syndromic cleft, accounting for 2% of all OFC (Rizos and Spyropoulos 2004). Popliteal pterygium syndrome (PPS) (OMIM:119500) is a rare autosomal dominant disorder affecting 1/300,000 people worldwide (Froster-Iskenius 1990) and in most cases is allelic, but more severe form of VWS. VWS/PPS are unique amongst syndromic OFC; in which cleft can either be of the lip or of the palate in the same pedigree.

Mutations in the *IRF6* gene (OMIM:607199) have been reported worldwide to cause VWS and PPS (Kondo et al. 2002; De Lima et al. 2009; Birkeland et al. 2011; Miñones-Suárez et al. 2012; Salahshourifar et al. 2012; Leslie et al. 2013; Malik et al. 2013; Tan et al. 2013).To date, about 300 mutations causing VWS and PPS have been reported in different populations across the world (Leslie et al. 2013) and these include missense, nonsense, and frameshift, microdeletions and splice-site mutations. These mutations are nonrandomly distributed and observed to be enriched in exon 4, which is in the highly conserved DNA-binding domain and in exon 7 and 9, which are in the less-conserved protein-binding domain (De Lima et al. 2009; Leslie et al. 2013). None of the 300 mutations previously reported included cases from sub-Saharan Africa and there is no information on the genetic causes of VWS in this population. In order to investigate the genetic causes of VWS in sub-Saharan African population, we obtained saliva samples from eight families with VWS and one PPS from Nigeria and Ethiopia for Sanger sequencing of the most commonly affected exons in *IRF6* (3, 4, 7, and 9).

## Methods

### Samples

The samples used for this genetic study were obtained as part of a larger study on genetics of orofacial clefts (Nigerian Craniofacial Anomalies Research Network [NigeriaCRAN] and Ethiopia Craniofacial Anomalies Research Network [EthiopiaCRAN]). Ethical approval was obtained from the Institutional Review Boards at the Lagos University Teaching Hospital, Lagos (IRB approval number: ADM/DCST/HREC/VOL.XV/321), Obafemi Awolowo University Teaching Hospital Ile-Ife (IRB approval number: ERC/2011/12/01), and the Addis Ababa University (IRB approval number: 003/10/surg). Written informed consent was obtained from every family recruited. We collected samples from affected proband, both parents, and from available affected and unaffected relatives. The Oragene collection kit (http://www.dnagenotek.com) was used to collect saliva samples from adults and children. For the newborn and children who were unable to spit, we used saliva sponges to soak saliva sublingually or in the buccal sulcus and these sponges were then placed into the Oragene collection kits.

The samples were then shipped to the Murray Laboratory in Iowa, for DNA processing and sequencing. The protocol for DNA processing is available on the Murray Lab website (http://genetics.uiowa.edu/protocols.php). The DNA samples concentration was measured using a Qubit 2.0 Fluorometer (http://www.invitrogen.com/site/us/en/home/brands/Product-Brand/Qubit.html) and a 4 ng/μL was used for Sanger sequencing.

### Clinical information

For a diagnosis of VWS to be made, lower lip pits must be present in one or several members of a family in addition to cleft of the lip and/or palate. The diagnosis of PPS is made when features of VWS are present in a family in addition to a popliteal web in the popliteal fossa.

**Figure 1:**
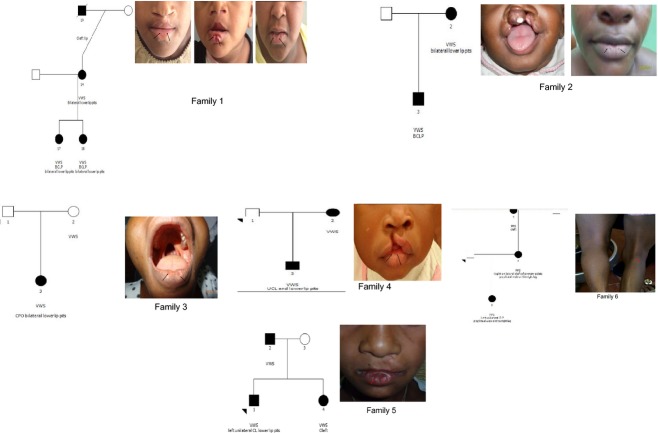
Pedigrees of the three VWS and PPS families. The black arrows point to the bilateral lower lip pits in the VWS families and popliteal web in the right popliteal fossa in the PPS family.

The nine families studied were as follows (Fig. 1):

Family 1: Patient 1 had complete bilateral cleft of the lip and palate (BCLP) and bilateral lower lip pits that has been repaired. Patient 2 had complete BCLP and unrepaired bilateral lower lip pits which appear as two distinct bulges. Patient 3 (mother) has unrepaired bilateral lower lip pits which are shallow and phenotypically different from the lip pits in patient two. Patient 4 (deceased maternal granddad) had a cleft lip (CL).

Family 2: Patient 1 had BCLP and Patient 2 (mother) has bilateral lower lip pits which are shallow and without a sinus.

Family 3: Patient 1 had cleft of the hard palate only (CPO) and bilateral lower lip pits which are shallow and without a sinus. Both parents appear clinically normal.

Family 4: Patient 1 had right incomplete cleft of the lip. Both parents appear clinically normal.

Family 5: Patient 1 had left incomplete cleft of the lip and bilateral lower lip pits. The elder sister was reported to have cleft with no specific details on the type. The father appears clinically normal.

Family 6: Patient 1 had complete left unilateral CLP and popliteal web on the right leg. Patient 2 (mother) had right unilateral cleft of the primary palate and popliteal web on the right leg. Patient 3 (maternal grandmother) was reported to have cleft with no specific details on the type.

### Sequencing

The method used for sequencing has been previously reported (Butali et al. 2013). In summary, we used the primer sets that were designed by De Lima et al. (2009) to sequence all the exons in the *IRF6* gene (NM_006147.2). A master mix containing 10 × NH4 buffer, 5% dimethyl sulphide (DMSO), 200 *μ*mol/L deoxyribonucleotide triphosphates (DNTPs), 50 *μ*mol/L MgCl, water, 20 *μ*mol/L of forward and reverse primers, and the 5 U/*μ*L Taq polymerase enzyme was prepared. We added 9 *μ*L of the master mix to 1 *μ*L of DNA in a 96-well plate. Two Centre d'Etude du Polymorphisme Humain (CEPH) samples and two water samples were added as controls. The list of primers and annealing temperatures are available at the Murray laboratory (http://genetics.uiowa.edu/protocols.php). Amplified DNA products were sent to Functional Biosciences in Madison, Wisconsin (http://order.functionalbio.com/seq/index) for sequencing using an ABI 3730XL (http://www.appliedbiosystems.com/absite/us/en/home.html). Chromatograms were transferred to a Unix workstation, base-called with PHRED (http://www.phrap.org/phredphrapconsed.html) (v.0.961028), assembled with PHRAP (http://www.phrap.org/) (v. 0.960731), scanned by POLYPHRED (http://droog.gs.washington.edu/polyphred/) (v. 0.970312), and viewed with the CONSED program (http://www.phrap.org/consed/consed.html) (v. 4.0). In probands with rare functional variants, we sequenced samples from their parents and relatives to confirm mutations that are de novo or segregating in the family. We then compared the mutations found in this study to mutations that are present in the African population and other populations in the 1000 genome (1KG) database (http://www.1000genomes.org/) and the two populations in the exome variant sequence (EVS) database (http://snp.gs.washington.edu/EVS/). All coding mutations were submitted into the locus-specific database (http://www.centralmutations.org/LsdbAdd.php).

We predicted the functional effects of these variants on the protein by using bioinformatics tools such as polyphen (http://genetics.bwh.harvard.edu/pph2/) (Adzhubei et al. 2010), SIFT (http://sift.jcvi.org/) (Kumar et al. 2009) and HOPE (http://www.cmbi.ru.nl/hope) (Venselaar et al. 2010).

## Results

**Figure 2:**
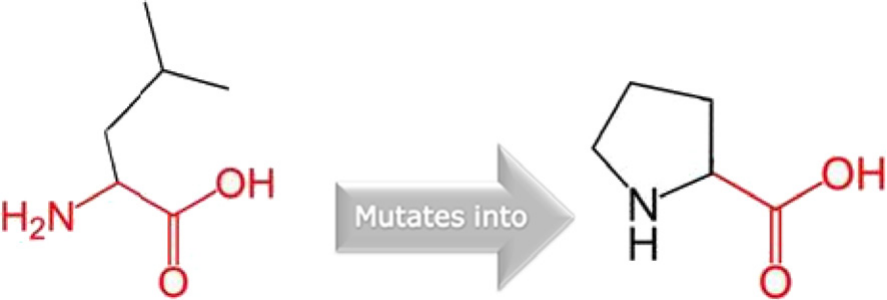
Schematic structures of the original leucine and the mutant (proline) at amino acid position 251. The backbone, which is the same for each amino acid, is colored red. The side chain, unique for each amino acid, is colored black.

**Figure 3:**
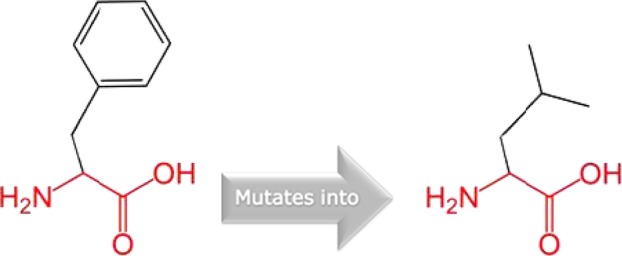
Schematic structures of phenylalanine into a leucine at position 230. The backbone, which is the same for each amino acid, is colored red and the side chain which is unique for each amino acid is colored black.

**Figure 4:**
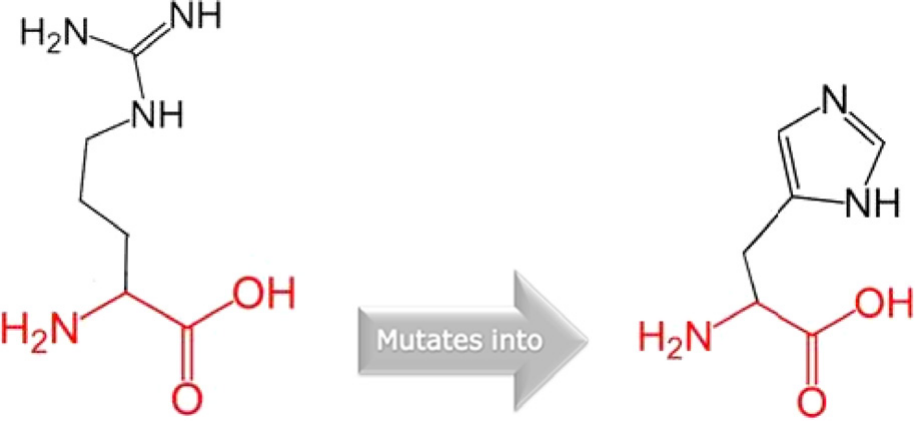
Schematic structures of an arginine into a histidine at position 84. The backbone, which is the same for each amino acid, is colored red and the side chain, unique for each amino acid, is colored in black.

We found six different heterozygous mutations (Figs. S1–S5) in the families investigated (Table 1). In family 1, we found a novel nonsense variant in exon 4 (p.Lys66X). We found a novel splice-site variant in exon 4 (p.Pro126Pro) in family 2. In family 3, we found a previously reported splice-site variant in exon 7 that changes the acceptor splice site. We found two variants in exon 7; a previously reported variant (p.Leu251Pro) (Figure 2) in family 4 and a novel variant (p.Phe230Leu) (Figure 3) in family 5. A previously known missense variant was found in exon 4 (p.Arg84His) (Figure 4) in family 6. All the mutations segregate in the families. Analyses using HOPE suggest that the proline introduced by the variant is a very rigid residue and this might abolish the required flexibility of the protein at this position. The residue is buried in the core of a domain and the differences between the wild-type and mutant residue might disturb the core structure of this domain. The mutant residue is smaller than the wild-type residue and the mutation will cause an empty space in the core of the protein.

**Table 1:** Showing families with VWS and PPS syndromes from Nigeria and Ethiopia, variants found and predicted functional effects on the protein

Families	Country	Diagnosis	DNA mutation^1^	Protein mutation^2^	Polyphen/SIFT predictions	1KG/EVS
1	Ethiopia	VWS	c.196A>T	p.Lys66X	Premature stop	0
2	Nigeria	VWS	c.551 T>A	Splice site		0
3	Nigeria	VWS	c.1061-2A>G	Splice site	Changes acceptor site	0
4	Nigeria	VWS	c.752T>C	p.Leu251Pro	Probably damaging/deleterious	0
5	Ethiopia	VWS	c.690T>G	p.Phe230Leu	Probably damaging/tolerated	0
6	Nigeria	PPS	rs121434227Gx>A	p.Arg84His	Probably damaging	0

1KG, 1000 genomes; EVS, exome variant server.

1 NM_006147.2

2 NP_006138

HOPE reveals that in the 3D structure, the wild-type residue is located in its preferred secondary structure, a *β*-strand and the mutant residue prefers to be in another secondary structure. Therefore, the local conformation will be slightly destabilized. The residue is buried in the core of a domain and the differences between the wild-type and mutant residue might disturb the core structure of this domain. The mutant residue is smaller than the wild-type residue and this will cause an empty space in the core of the protein.

HOPE suggests that the wild-type residue is bigger and was positively charged and the mutant residue is neutral and smaller. This means that the charge of the buried wild-type residue is lost by this mutation. The residue has DNA interactions or is located in a DNA-binding region and the differences in the properties between wild-type and mutant residue can easily cause loss of these interactions or disturb the domain that will affect the function of the protein. In addition, the mutant residue is located near a highly conserved position. Furthermore, the differences between the wild-type and mutant residue might disturb the core structure of this domain as the residue is buried in the core domain that will cause an empty space in the core of the protein. Therefore, this mutation is possibly damaging.

## Discussion

We found six different mutations in VWS and PPS families from two populations in sub-Sahara. The nonsense variant (p.Lys66X) is novel and never been previously reported, thus suggesting that it is specific to this population. This variant was found in exon 4 leading to the formation of a premature stop codon and truncation of the protein. This leads to haploinsufficiency of the *IRF6* gene; a previously described mechanism for VWS (Kondo et al. 2002). We also found a novel synonymous variant in exon 4 that is 1 bp from the canonical donor splice site in intron 5. Scioletti et al. (2010) previously reported a splice-site variant in intron 4 that affects the acceptor site using the Human Splicing Finder (HSF v.2.4, http://www.umd.be/HSF/). The novel splice-site variant in this study is not predicted to have any effect on splicing when we conducted bioinformatic analysis using the HSF. The splice-site variant in intron 7 was previously reported in a Brazilian Family (De Lima et al. 2009).

The missense mutation (p. Leu251Pro) was previously reported in a Belgian family (Ghassibe et al., 2004). HOPE analyses of the missense variant suggest that Proline; a rigid residue abolishes the flexibility of IRF6 protein at this position leading to an altered *IRF6* gene product. Therefore, p.Leu251Pro is a dominant negative mutation and will affect the molecular function of the gene. The dominant negative mechanism has been previously described for VWS and PPS syndromes (Kondo et al. 2002; Item et al. 2005). The novel missense variant (p.Phe230Lue) is a dominant negative mutation that acts antagonistically to the wild type leading to disturbances in the conformity of the protein. The missense variant found in the PPS family (p. Arg84His) is also a dominant negative mutation and one of the two major mutations that are frequently reported for PPS (De Lima et al. 2009; Leslie et al. 2013). This study provides data on the geographical spread of the variant and role in PPS etiology.

In three of the families with VWS, we did not find any mutation in the *IRF6* gene. This is not surprising as 25% of families with VWS do not have a mutation in the *IRF6* gene (De Lima et al. 2009). It is possible that mutations at a different locus or loci account for the etiology of VWS in these families. A recent study identified a second locus in chromosome 1 through linkage and exome sequencing studies (Peyrard et al. 2013). Fine mapping of this region using Sanger sequencing suggests that mutations in the grainy-head like three (*GRHL3*) gene contributes to 3–5% of cases with VWS. In the remaining 20% of cases, it is possible that there may be large deletions that take out the IRF6 gene or other regions in the genome. Exome sequencing of these samples is planned and this will unravel additional etiological locus or loci involved.

The different lip phenotypes observed in this study (e.g., lip pits and lip bulbs) suggest that there are major and minor lip phenotypes in VWS. This observation is consistent with the lip phenotypes described in previous studies (Desmyter et al. 2010; Salahshourifar et al. 2012). Furthermore, three of the individuals with VWS and the individual with PPS were initially diagnosed as nonsyndromic following clinical examination. However, mutation screen in the *IRF6* gene confirmed these individuals to be VWS and PPS, respectively. Therefore, it is essential for all previously described cases of nonsyndromic clefts to be examined for these minor VWS phenotypes and mutations in the *IRF6* gene.

Our data confirm the presence of *IRF6*-related VWS and PPS in sub-Saharan Africa and highlight the importance of screening for novel mutations in known genes when studying diverse global populations. Screening other exons of the gene may be useful, especially if the most commonly affected exons show no mutation. This is important for counseling and prenatal diagnosis for high-risk families, especially in Africa where there are limited genetic counselors.

## Web Resources

http://www.omim.org; genetics@uiowa.edu; http://order.functionalbio.com/seq/index; http://www.umd.be/HSF/; http://www.1000genomes.org/; http://snp.gs.washington.edu/EVS/;http://www.centralmutations.org/LsdbAdd.php; http://genetics.bwh.harvard.edu/pph2/; http://sift.jcvi.org/; http://www.cmbi.ru.nl/hope

## Supporting Information

**Figure S1.** Chromatogram showing a single-nucleotide change from A to C at position 498 in exon 7. This nucleotide change led to a missense mutation (p.Phe230Leu). **Figure S2.** Chromatogram showing a single-nucleotide change from T to A at position 290 in exon 4. This nucleotide change led to a nonsense mutation (p.Lys66X). **Figure S3.** Chromatogram showing a single-nucleotide change from T to C at position 348 2 base pairs from exon 8. This nucleotide change led to the creation of a potential new acceptor splice site in exon 8. **Figure S4.** Chromatogram showing a single-nucleotide change from A to G at position 424 in exon 7. This nucleotide change led to a missense mutation (p. Leu251Pro). **Figure S5.** Chromatogram showing a single-nucleotide change from A to G at position 247 in exon 4. This nucleotide change led to a missense mutation (p. Arg84His).
