# Supplementary material for: Novel IRF6 mutations in families with Van Der Woude syndrome and popliteal pterygium syndrome from sub-Saharan Africa
Source: Mol Genet Genomic Med. 2014 Jan 27;2(3):254–60. doi: 10.1002/mgg3.66 (PMC4049366; doi:10.1002/mgg3.66)
Supplement: Supplementary file 1 [file mgg30002-0254-SD1.pdf]

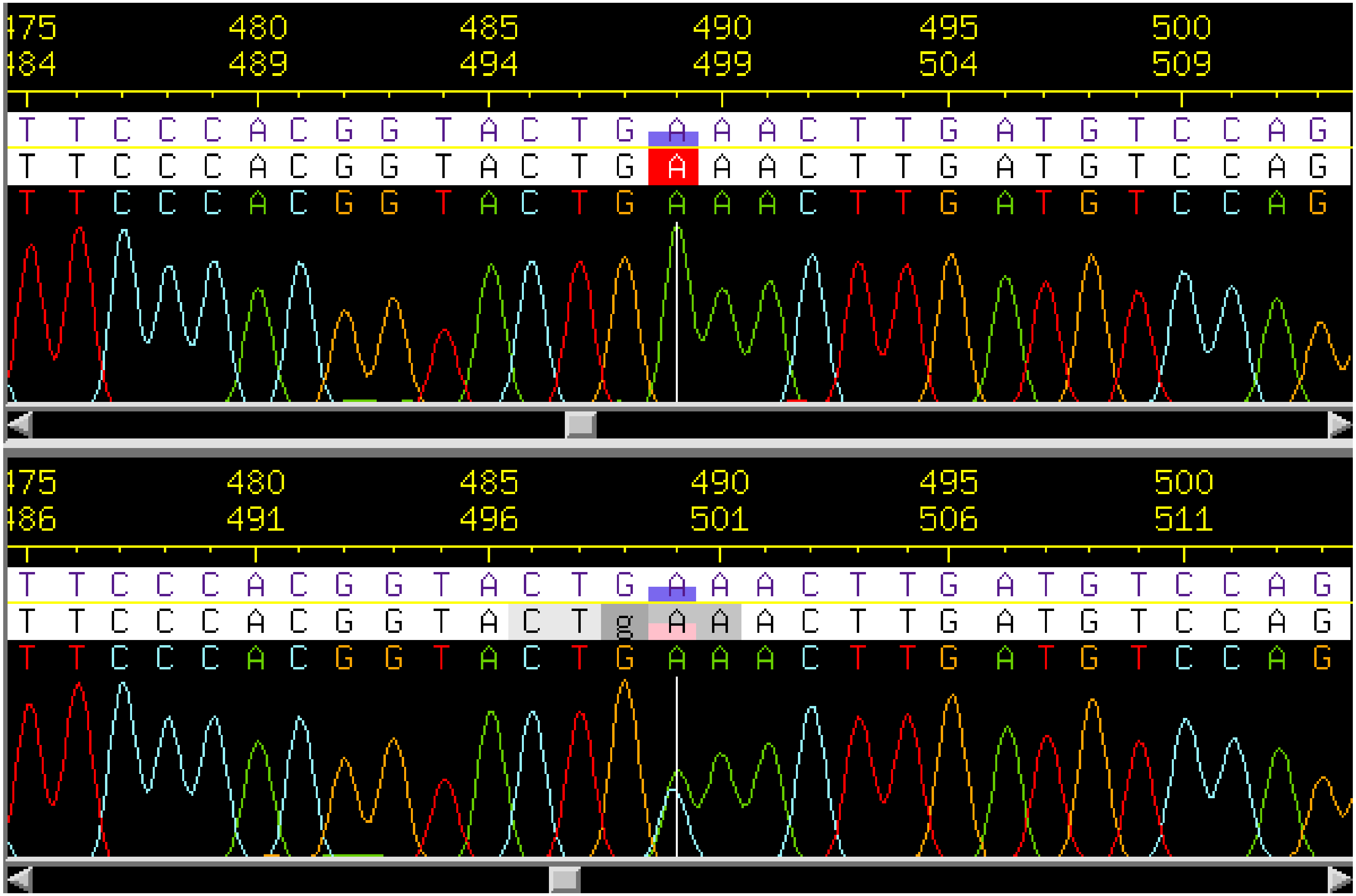

Supp. Figure S1.Chromatogram showing a single nucleotide change from A to C at position 498 in exon 7. This nucleotide change led to a missense mutation (p.Phe230Leu).

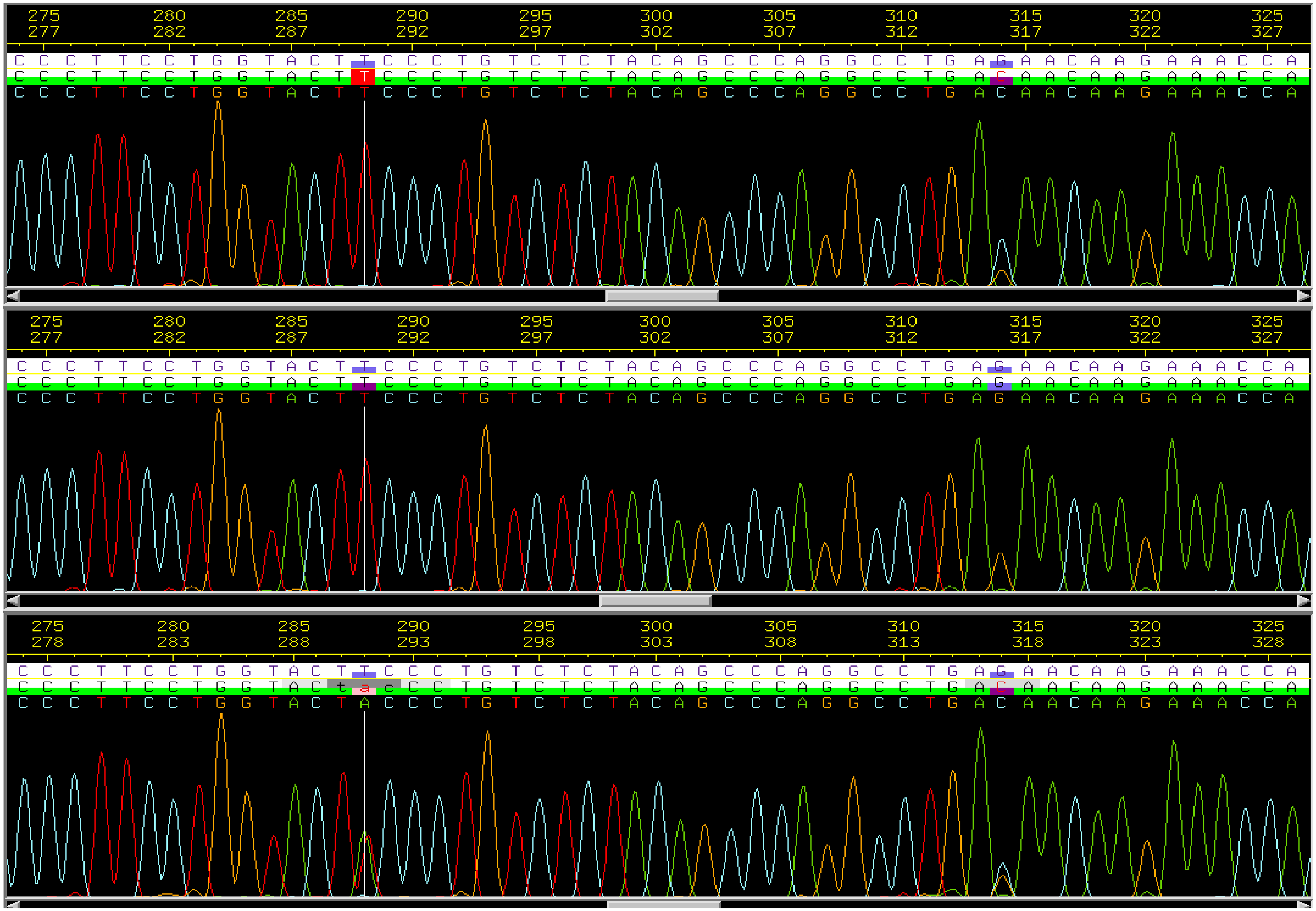

Supp. Figure S2.Chromatogram showing a single nucleotide change from T to A at position 290 in exon 4. This nucleotide change led to a nonsense mutation (p.Lys66X).

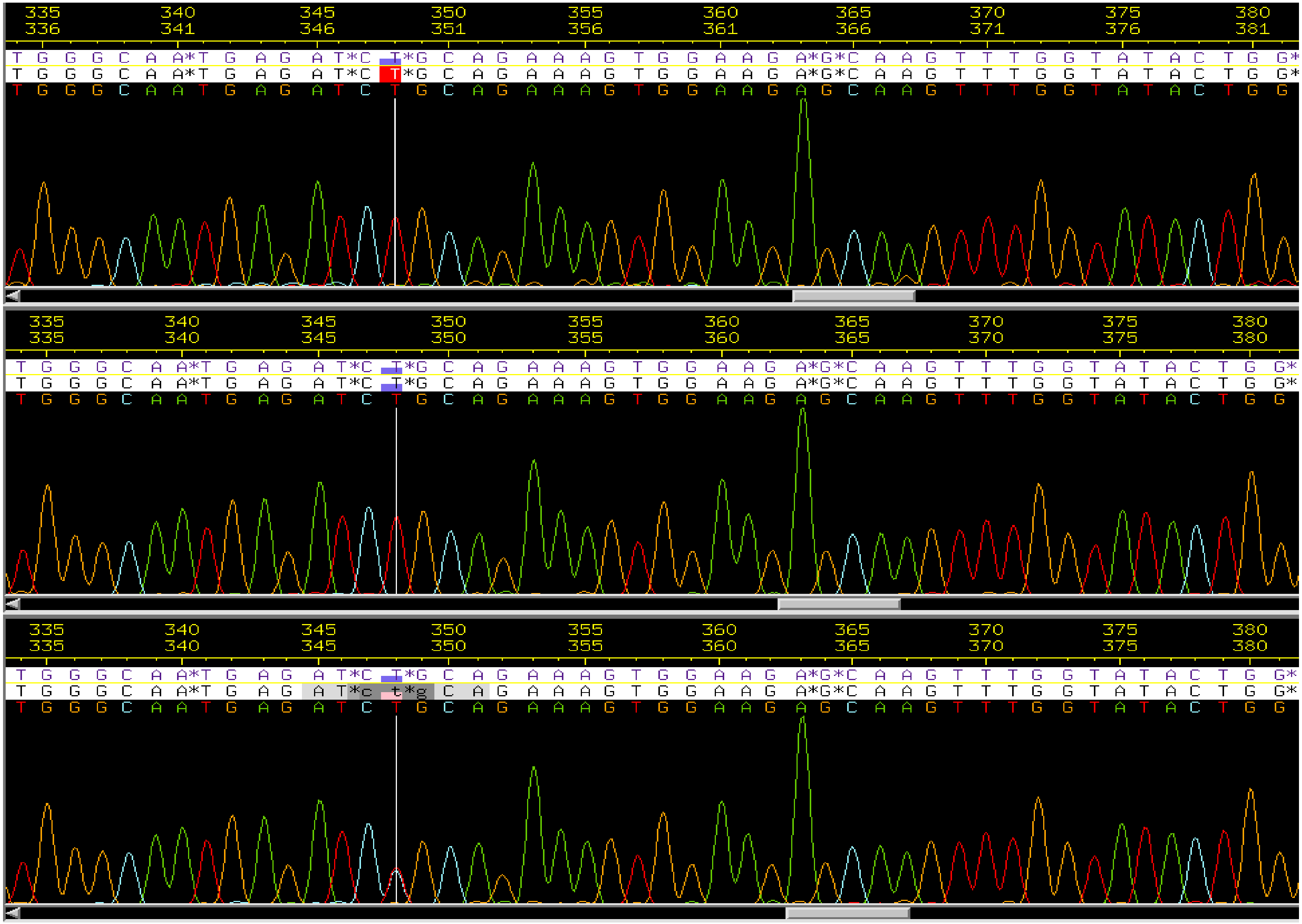

Supp. Figure S3. Chromatogram showing a single nucleotide change from T to C at position 348 2 base pairs from exon 8. This nucleotide change led to the creation of a potential new acceptor splice site in exon 8.

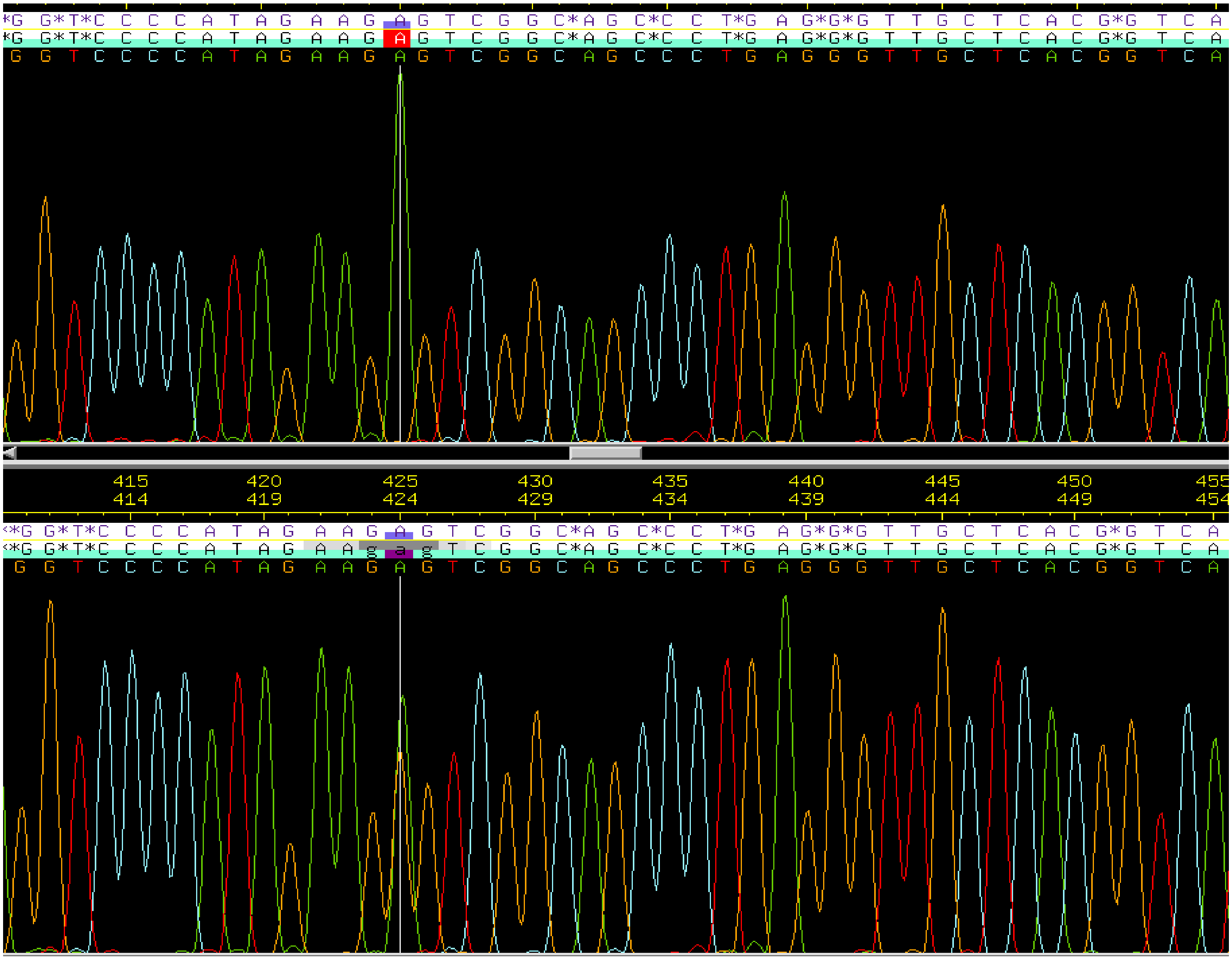

Supp. Figure S4.Chromatogram showing a single nucleotide change from A to G at position 424 in exon 7. This nucleotide change led to a missense mutation (p. Leu251Pro).

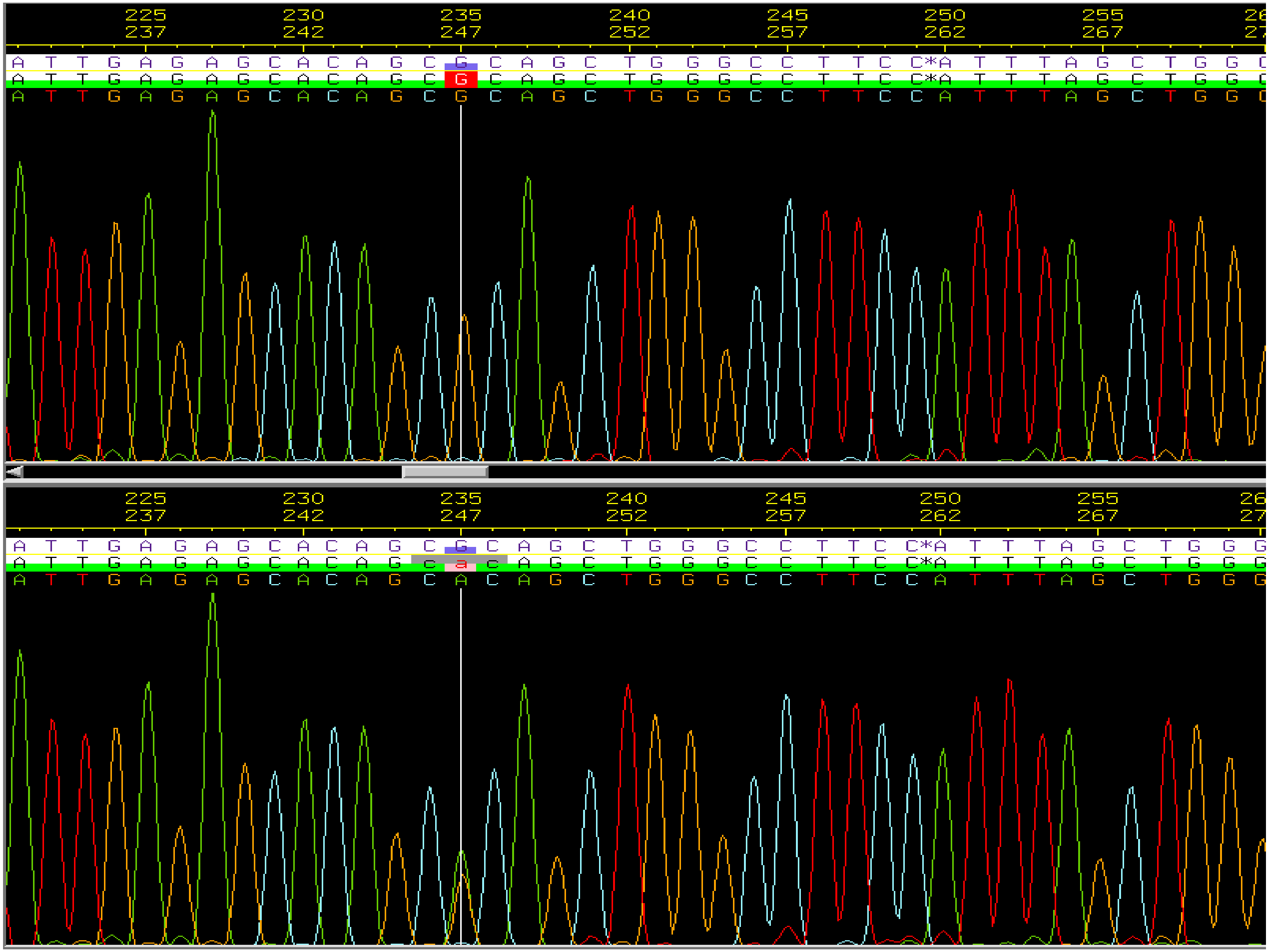

Supp. Figure S5. Chromatogram showing a single nucleotide change from A to G at position 247 in exon 4. This nucleotide change led to a missense mutation (p. Arg84His).
